# Prognostic effects of clinical and CT imaging features on critically ill patients with interstitial lung disease hospitalized in respiratory intensive care unit

**DOI:** 10.1038/s41598-019-53865-0

**Published:** 2019-11-20

**Authors:** Shenyun Shi, Yonglong Xiao, Xiaohua Qiu, Yan Li, Yuying Qiu, Kefeng Zhou, Hourong Cai

**Affiliations:** 1 Department of Respiratory Medicine, Nanjing Drum Tower Hospital, Clinical College of Nanjing Medical University, Nanjing, 210008 Jiangsu China; 2 Department of Respiratory Medicine, Nanjing Drum Tower Hospital, Nanjing University Medical School, Nanjing, 210008 Jiangsu China; 3 Department of Radiology, Nanjing Drum Tower Hospital, Nanjing University Medical School, Nanjing, 210008 Jiangsu China

**Keywords:** Risk factors, Signs and symptoms

## Abstract

The study aimed to evaluate the clinical and imaging features of critically ill patients with interstitial lung disease (ILD) treated in respiratory intensive care unit (RICU) and assess the prognostic effects of these factors. A total of 160 severe ILD patients admitted to the RICU were finally enrolled in this study. The clinical, imaging and follow-up data of them were studied retrospectively. The in-hospital mortality and total mortality were 43.1% and 63.8% respectively. By multivariate cox regression analysis, shock (OR = 2.39, P = 0.004), pulmonary fibrosis on CT (OR = 2.85, P = 0.002) and non-invasive ventilation (OR = 1.86, P = 0.037) were harmful factors to survivals of critically ill patients with ILD. In contrast, oxygenation index (OR = 0.99, P = 0.028), conventional oxygen therapy (OR = 0.59, P = 0.048) and β-lactam antibiotics use (OR = 0.51, P = 0.004) were protective factors. There is significant difference of survivals between patients with and without fibrosing ILD on CT (Log-rank, p = 0.001). The prognosis of critically ill patients with ILD was poor. Shock, respiratory failure and fibrosing signs on chest CT affected the prognosis. Chest CT was considered as a valuable tool to indicate the prognosis.

## Introduction

Interstitial lung disease (ILD) is a group of heterogeneous lung diseases with pulmonary alveolar unit inflammation or interstitial fibrosis. ILD could be with specific etiology (connective tissue disease, environmental exposure, drugs, etc) or idiopathic^1^. Severe ILD can be life-threating and cause high mortality even though hospitalized in intensive care unit (ICU). There had been few studies on the clinical characteristics and survival analysis of critically ill ILD patients. One study reported a hospital mortality rate about 66% in patients with ILD requiring critical treatment in ICU^2^. To better understand the prognostic factors that can be evaluated in severe ILD patients, the clinical and imaging features of patients with severe ILD hospitalized in respiratory intensive care unit (RICU) were retrospectively reviewed.

## Materials and Methods

### Clinical data

During the period from April 2013 to June 2018, a total of 343 patients with the diagnoses of “interstitial lung disease” or “diffuse pulmonary disease” hospitalized in RICU of Nanjing drum tower hospital were reviewed in detail. This study was consented by Ethics Committee of Nanjing Drum Tower Hospital. The Ethics Committee waived the need for informed consent as the study was retrospective and the data were analyzed anonymously. Patients with the final primary diagnosis of infective or tumorous pulmonary diffuse lesions were excluded, with 319 eligible cases which presented with severe respiratory flares requiring intensive management. There were 273 cases with complete clinical data and HRCT images. Through telephone follow-up, 160 cases had complete follow-up results. The main data collected included demographic features, rheumatic signs, acute renal injury (an increase of serum creatinine ≥0.3 mg/dl within 48 hours, or an increase in serum creatinine to ≥1.5 times baseline), shock (indifferent mind, unresponsive, pale skin rapid shallow breathing, systolic blood pressure below 90 mmHg and oliguria), chest high resolution CT (HRCT) and treatment modality in RICU. Survival was assessed up to September 01, 2018. Survival status was identified by reviewing the medical records or telephone follow-ups.

## Methods

The diagnosis of ILD was mainly based on clinical, imaging and pathological data, and the specific diagnostic criteria referring to the guidelines of interstitial lung disease published by British thoracic society in 2008^3^ and the 2013 American Thoracic Society/European Respiratory Society consensus classification update of the idiopathic interstitial pneumonias^4^. The clinical characteristics of 160 enrolled patients including laboratory data and treatment methods were analyzed in this study. All patients with chest HRCT data were adjusted to the PACS system data storage, and only the CT data before RICU admission were analyzed. According to the study of the Fleischner Society^5^, the abnormalities on HRCT were divided into ground-glass opacity, reticular and honeycomb. The extent of each abnormality was quantified for the whole lung. Based on the study conducted by Akira *et al*.^6^, the lungs were divided into 6 lung zones (upper zone: above the level of the tracheal carina, lower zone: below the level of the inferior pulmonary vein, middle zone: the area of the lung between the upper and lower zones). The overall extent of lung involvement was calculated by each of six lung zone values. Rang score was referred to Silva grading system^7^: 0 point: absent; 1 point: 1% <focus range ≤4% of whole lung; 2 point: 5%< focus range ≤25%; 3 point: 26%< focus range ≤50%; 4 point: >50% of whole lung. In this study, 76 ILD patients were classified into two groups based on the range score of honeycomb. The non-fibrosing group included grades 0–1 and fibrosing group consisted of grades 2–4. All chest CT images were analyzed by a professional thoracic radiologist and a senior respiratory clinician. If they had different opinions on CT images, they made the decisions after discussions.

### Statistical analysis

Survival was calculated as the time from RICU admission to death. Patients who were alive at the last contact were censored. Log-rank test was used to compare survival differences between two groups without or with pulmonary fibrosis on HRCT. For data with normal distribution, means and standard deviations were used for descriptive statistics; and for data with non-normal distribution, medians and interquartile ranges. Group differences were tested by t-tests or X^2^ tests. The independent prognostic role of variables were evaluated by multivariate cox regression analysis. All analyses were performed with SPSS software, version 23.0 (SPSS, Inc., Chicago, IL, USA). All tests were two-sided and performed at a significance level of 0.05.

### Ethics approval

This study was consented by Ethics Committee of Nanjing Drum Tower Hospital. The Ethics Committee waived the need for informed consent as the study was retrospective and the data were analyzed anonymously.

## Results

### Prognostic effects of baseline clinical characteristics on critically ILD patients

In this study, 160 critically ill ILD patients were enrolled who had clinical and follow-up data. The mean length of their hospitalization in RICU was 12.3 days. The death rate in the RICU was 43.1%, and respectively the total death rate was 63.8% according to the final follow-up. The baseline clinical characteristics of 160 critically ill ILD patients which might be related with their prognosis were listed in Table 1. We found that clubbed-finger, arthralgia, shock and proteinuria were associated with survival status. By multivariate Cox proportional regression analysis, shock (OR = 2.39, P = 0.004) was found be an independent prognostic factor in critically ill patients with ILD.

**Table 1:** Baseline clinical characteristics of 160 critically ill ILD patients.

	survivors (n = 58)	nonsurvivors (n = 102)	P value
Age in years	69.0 ± 12.8	66.8 ± 11.7	0.254
Female	19 (32.8%)	33 (32.3%)	0.958
Current smoker	22 (37.9%)	38 (37.3%)	0.932
Known cardiovascular disease	21 (36.2%)	24 (23.5%)	0.086
Snoring or popping sound	53 (91.4%)	98 (96.1%)	0.215
Clubbed-finger	4 (6.9%)	1 (0.10%)	0.039
Skin rash	8 (13.8%)	18 (17.6%)	0.525
Arthralgia	3 (5.2%)	0 (0.0%)	0.020
Shock	2 (3.4%)	24 (23.5%)	0.001
Acute kidney injury	5 (8.6%)	13 (12.7%)	0.427
Proteinuria	12 (21.1%)^a^	40 (40.8%)^b^	0.012

a: n = 57, b: n = 98.

### Prognostic effects of biochemical characteristics on critically ILD patients

Statistically significant relationship between the survival status and biochemical results including white blood cell count, c-reactive protein, immunoglobulin M, aspartate aminotransferase, lactate dehydrogenase, blood lymphocytes count, CD4+ lymphocytes count, CD8+ lymphocytes count, cytokeratin 21-1, oxygenation index was analyzed.The detailed relationship between prognosis and serological variables was shown in Table 2. In the multivariate cox regression analysis, oxygenation index (OR = 0.99, P = 0.028) was an independent and protective factor for prognosis of these 160 critically ill ILD patients. The detailed results of Cox regression analysis of the remaining indicators were shown in Table 3.

**Table 2:** Analyses of the associations between serological variables and survival status in 160 critically ill ILD patients.

Variables	survivors (n = 58)	nonsurvivors (n = 102)	P value
WBC (*10^9^)	10.0 (7.5–12.0)	10.1 (7.0–13.8)	0.619
N (%)	85.5 (72.6–89.4)	88.3 (80.1–92.3)	0.016
Hb (g/l)	124.5 (107.0–137.3)	125.0 (116.8–142.0)	0.558
PLT (*10^9^)	218.0 (181.3–284.0)	176.5 (121.0–259.3)	0.002
L (*10^9^)	0.97 (0.66–1.39) (n = 53)	0.73 (0.45–1.13) (n = 75)	0.021
CD4+ (*10^6^)	315.5 (186.8–486.5) (n = 54)	189.0 (106.0–368.0) (n = 77)	0.004
CD8+ (*10^6^)	270.5 (148.3–474.3) (n = 54)	183.0 (91.5–388.5) (n = 77)	0.040
PCT (ng/ml)	0.05 (0.02–0.12) (n = 45)	0.08 (0.04–0.27) (n = 66)	0.053
CRP (mg/l)	27.6 (6.9–59.9) (n = 57)	47.8 (9.4–94.6) (n = 101)	0.048
IgA (g/l)	2.6 (1.8–3.3) (n = 53)	2.7 (2.0–3.8) (n = 89)	0.546
IgG (g/l)	12.2 ± 4.2(n = 53)	12.3 ± 4.6(n = 89)	0.960
IgM (g/l)	1.2 (0.9–1.9) (n = 53)	1.0 (0.7–1.4) (n = 89)	0.013
IgE (IU/ml)	102.0 (44.0–222.0) (n = 51)	100.0 (60.0–167.5) (n = 73)	0.974
AST (U/L)	21.3 (15.5–27.2) (n = 57)	27.5 (19.4–45.6) (n = 101)	0.007
ALT (U/L)	22.7 (13.8–36.3) (n = 57)	24.9 (17.2–40.1) (n = 101)	0.212
LDH (U/L)	343.0 (279.0–438.0) (n = 57)	456.0 (310.5–603.0) (n = 101)	0.001
Scr (umol/l)	57.5 (49.8–73.7)	57.0 (46.7–70.2)	0.306
CYF 21-1 (ng/ml)	5.2 (3.9–8.0) (n = 51)	11.0 (7.4–16.6) (n = 89)	0.000
NSE (ng/ml)	15.9 (12.6–22.8) (n = 51)	26.5 (18.7–38.3) (n = 89)	0.000
CEA (ng/ml)	3.1 (2.2–4.2) (n = 51)	6.7 (3.1–12.2) (n = 89)	0.000
PaO2 (mmHg)	74.0 (63.0–89.0) (n = 55)	67.0 (54.8–79.0) (n = 101)	0.014
PaCO2 (mmHg)	37.4 (32.5–43.5) (n = 55)	35.2 (30.6–40.0) (n = 100)	0.106
P/F (mmHg)	230.4 ± 83.1(n = 55)	173.2 ± 79.8(n = 101)	0.000
ANA	29 (54.7%) (n = 53)	34 (37.8%) (n = 90)	0.049
ENA	32 (60.4%) (n = 53)	44 (48.9%) (n = 90)	0.184
ANCA	5 (9.4%) (n = 53)	9 (10.0%) (n = 90)	0.912
G test	4 (8.2%) (n = 49)	19 (27.5%) (n = 69)	0.009
GM test	1 (2.0%) (n = 49)	6 (8.6%) (n = 70)	0.136

Variables: WBC White blood cell count; N Percentage of neutrophil; Hb Hemoglobin; PLT Platelet; L Blood lymphocyte count; CD4 + CD4 + lymphocytes; CD8 + CD8 + lymphocytes; PCT Procalcitonin; CRP C-reactive protein; IgA Immunoglobulin A; IgG Immunoglobulin G; IgM Immunoglobulin M; Ig E Immunoglobulin E; AST Aspartate aminotransferase; ALT Alanine aminotransferase; LDH Lactate dehydrogenase; Scr Serum creatinine; CYF 21-1 Cytokeratin 21-1; NSE Neuron specific enolase; CEA Carcinoembryonic antigen; PaO2 Oxygen partial pressure; PaCO2 Carbon dioxide partial pressure; P/F Oxygenation index; ANA Autoantibody ANA positive; ENA Autoantibody ENA positive; ANCA Anti-neutrophil cytoplasmic antibody positive; G test Fungal glucan test positive; GM test Galactomannan test positive.

**Table 3:** Cox proportional-hazards regression of serological variables of 160 critically ill ILD patients.

Variables	P value	OR	95.0% CI for Exp(B)	
			Lower	Upper
N (%)	0.705	0.993	0.957	1.03
PLT (*10^9^)	0.765	1.001	0.996	1.005
CRP (mg/l)	0.713	0.999	0.996	1.003
IgM (g/l)	0.487	0.869	0.586	1.29
ANA	0.792	1.1	0.541	2.239
AST (U/L)	0.205	0.991	0.976	1.005
LDH (U/L)	0.086	1.002	1	1.004
L (*10^9^)	0.777	1.248	0.269	5.801
CD4+ (*10^6^)	0.255	0.998	0.996	1.001
CD8+ (*10^6^)	0.69	1	0.999	1.002
G test	0.256	1.661	0.693	3.984
CYF 21-1 (ng/ml)	0.938	0.998	0.951	1.048
NSE (ng/ml)	0.16	1.021	0.992	1.051
CEA (ng/ml)	0.468	1.003	0.995	1.011
PaO2 (mmHg)	0.866	1.002	0.98	1.024
P/F (mmHg)	0.028	0.993	0.987	0.999

Variables: N Percentage of neutrophil; PLT Platelet; CRP C-reactive protein; IgM Immunoglobulin M; ANA Autoantibody ANA positive; AST Aspartate aminotransferase; LDH Lactate dehydrogenase; L Blood lymphocyte count; CD4 + CD4 + lymphocytes; CD8 + CD8 + lymphocytes; G test Fungal glucan test positive; CYF 21-1 Cytokeratin 21-1; NSE Neuron specific enolase; CEA Carcinoembryonic antigen; PaO2 Oxygen partial pressure; P/F Oxygenation index

### Prognostic effects of treatment aspects on critically ILD patients

In this study, main treatments included the following aspects: oxygen therapy (nasal catheter oxygen therapy, mask oxygen therapy, non-invasive ventilation, invasive ventilation), glucocorticoid, immunosuppressive agent, anti-infection (covering bacteria, viruses and fungi), sedative therapy. In 58 patients who survived during the follow-up, 55.2% of the patients accepted high doses of glucocorticoid (>1 mg/kg/d). In 102 patients with severe ILD who died during follow-up, 72.5% of the patients accepted high doses of glucocorticoid. The main antibiotics used during hospitalization include, β-lactams, carbapenems, sulfonamides, aminoglycosides, glycopeptide, and macrolides. The immunosuppressants used mainly include tacrolimus, tripterygium, hydroxychloroquine, cyclosporine, and cyclophosphamide. The relationship between treatments aspects and survival status in 160 critically ill ILD patients was shown in Table 4. By multivariate Cox proportional regression analysis, non-invasive ventilation (OR = 1.86, P = 0.037) was harmful factor to survival of critically ill patients with ILD. Respectively, conventional oxygen therapy (OR = 0.59, P = 0.048) and β-lactam antibiotics use (OR = 0.51, P = 0.004) were protective factors. The results of multivariate Cox proportional regression analysis of other factors was shown in Table 5.

**Table 4:** Analyses of the associations between treatment aspects and survival status in 160 critically ill ILD patients.

	survivors (n = 58)	nonsurvivors (n = 102)	P value
Immunosuppressive agents	25 (43.1%)	33 (32.4%)	0.174
Conventional oxygen therapy	53 (96.4%)^b^	70 (69.3%)^c^	0.000
Non-invasive ventilator	12 (21.8%)^b^	64 (63.4%)^c^	0.000
Invasive ventilator	0 (0.0%)^b^	12 (11.9%)^c^	0.008
Sedative	14 (24.1%)	66 (64.7%)	0.000
High-dose glucocorticoid (>1 mg/kg/d) therapy	32 (55.2%)	74 (72.5%)	0.025
Beta lactam antibiotic	50 (86.2%)	67 (65.7%)	0.005
Carbapenem	20 (34.5%)	62 (60.8%)	0.001
Sulfonamide	32 (55.2%)	68 (66.7%)	0.149
Quinolones	20 (34.5%)	29 (28.4%)	0.425
Glycopeptide	1 (1.7%)	13 (12.7%)	0.018
Macrolides	0 (0.0%)	2 (2.0%)	0.283
Antiviral therapy	36 (62.1%)	69 (67.6%)	0.475
Antifungal therapy	21 (36.2%)	58 (56.9%)	0.012

b: n = 55, c: n = 101.

**Table 5:** Cox proportional-hazards regression of treatment aspects of 160 critically ill ILD patients.

	P value	OR	95.0% CI for Exp(B)	
			Lower	Upper
Conventional oxygen therapy	0.048	0.591	0.351	0.995
Non-invasive ventilator	0.037	1.855	1.039	3.311
Invasive ventilator	0.261	1.471	0.75	2.885
Sedative	0.648	1.147	0.636	2.068
High-dose glucocorticoid (>1 mg/kg/d) therapy	0.434	1.206	0.754	1.929
Beta lactam antibiotic	0.004	0.509	0.32	0.81
Carbapenem	0.982	0.994	0.619	1.598
Glycopeptide	0.557	1.219	0.629	2.364
Antifungal therapy	0.329	1.252	0.797	1.967

### Prognostic effects of CT imaging features on critically ILD patients

**Figure 1:**
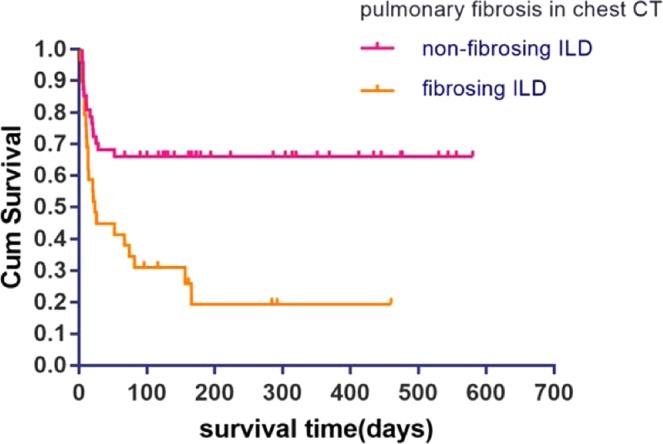
The respective Kaplan-Meier curve of critical patients with fibrosing ILD and non-fibrosing ILD. Log-rank test (P = 0.001).

Among 160 patients with severe ILD with clinical and follow-up data, 76 patients had radiographic data. Based on the range score of honeycomb, 76 patients with severe ILD were divided into fibrosing group (29 patients) and non-fibrosing group (47 patients). The survival curve of the two groups is shown in Fig. 1. The difference of survival rate between the two groups was statistically significant after the Log-range (p = 0.001). By Cox proportional regression analysis, we found that pulmonary fibrosis on HRCT (OR = 2.85, p = 0.002) was an independent prognostic factor for patients with severe ILD.

## Discussion

In this study, shock was identified to be an independent risk factor for the survival of patients with severe ILD. In patients with severe ILD with shock, various cytokines were activated, especially tumor necrosis (TNF), lung tissue nuclear factor-κB (NF-kB). These cytokines are widely involved in the body’s immune response, immune response gene transcription regulation, resulting in lung tissue damage^8^. Therefore, in the case of severe ILD patients with shock, anti-inflammatory treatment should be strengthened and intervention can be made against the cytokines mentioned above in order to reduce the acute damage caused by shock to lung tissue. This may improve the prognosis of patients with severe ILD.

As at the Berlin conference on acute respiratory distress syndrome (ARDS), experts believed that ARDS was an acute inflammatory lung injury, resulting in increased pulmonary vascular permeability. ARDS was different in degree and mortality, mild, moderate and severe ARDS were associated with increased mortality^9^. This finding was consistent with the finding of our study. The higher the oxygenation index, the higher the survival rate of ILD patients. Oxygenation index in patients with severe ILD could be an independent index and helped to judge the prognosis of patients.

About the treatment aspects, the results showed that common oxygen inhalation (nasal catheter, mask) and non-invasive ventilation were independent factors affecting the survival of patients with severe ILD. Common oxygen therapy is an independent protective factor for their survival while noninvasive ventilation is a risk factor. Mechanical ventilation is often needed in patients with severe ILD in ICU to improve the symptoms of dyspnea, but the risk of secondary infection increases^10^. There is no uniform standard for the choice of noninvasive and invasive ventilation. Gungor *et al*.^11^ through the analysis of 120 patients with ILD after admission to intensive care unit with invasive ventilation and non-invasive ventilation and mortality, finding an assessment of acute physiology and chronic health evaluation (APACHE II) score patients with severe illness rated <20, noninvasive ventilation is a better option. Invasive ventilation was not beneficial to the survival of patients with ILD with APACHE II score >20. According to Gaudry’s research^12^, although patients with ILD were admitted to hospital for invasive ventilation, most of them would still die within six months. However, invasive ventilation is of long-term value in patients with severe ILD and earned time for lung transplantation. The results of Molica *et al*.^13^ showed that mechanical ventilation had no significant improvement in the survival rate of patients with end-stage ILD. Therefore, the choice of mechanical ventilation in patients with severe ILD needs to consider a variety of comprehensive factors, including the wishes of patient and long-term expectation. In addition, the results of our study show that the use of β-lactams is an independent protective factor for the survival of patients with severe ILD patients, but there is no related report in the past. It has been suggested that antibiotics (in addition to sulfamethoxazole and macrolide drugs) may not contribute to the survival of patients with severe ILD^14^. Kawamura *et al*.^15^ reported that azithromycin was associated with improved prognosis in patients with severe ILD, suggesting that a combination of macrolide antibiotics might increase survival. But it is worth noting that there was no significant difference between the two groups of patients in our study. The result may be related to the use of macrolide antibiotics in only 2 patients with severe ILD in our study. Therefore, the relationship between the use of antibiotics and the survival of patients with ILD needs further clinical study.

Chest HRCT plays an important role in the diagnosis and monitoring of ILD. It not only provides a clear and noninvasive diagnosis of typical pulmonary diseases, and also provides a more accurate diagnostic basis for ambiguous cases^16^. The most common manifestation of ILD by HRCT is the ground glass, reticular, honeycomb and retraction bronchiectasis^17^. In this study, there is significant difference of survivals between patients with and without fibrosing ILD on CT. It is mentioned in the literature that chest CT pulmonary fibrosis is defined as a sign of retraction bronchiectasis and/or honeycomb changes on chest CT, which is usually a sign of severe end-stage ILD^18^. In the case of noninvasive ventilation, the pulmonary alveolar capillary gas exchange and lung compliance in these patients with ILD on chest CT showed fibrosis were worse than those on chest CT with non-fibrosis on chest CT^19^.

There are some limitations in this study. First of all, the study is a retrospective observation study in which the threshold of severe ILD admission to RICU is difficult to unify and treatment options are uneven. This study only describes the reality of ILD in RICU. Secondly, due to the lack of some clinical and pathological data, this study has not yet studied the patients with severe ILD by different subtype grouping. Finally, there is no standard quantitative tool to judge chest CT and the interpretation is subjective.

## Conclusions

Above all, the overall prognosis of patients with severe ILD is poor. Shock, respiratory failure and severity directly affect the prognosis. Chest CT is a valuable tool to indicate prognosis. The value of antibiotics in the treatment of ILD patients is worth further verification.

## Data Availability

Data can be submitted by corresponding authors in case of a request.

## References

[1] Zafrani L (2014). Acute respiratory failure in critically ill patients with interstitial lung disease. PLoS One.

[2] Gannon WD (2018). Outcomes and Mortality Prediction Model of Critically Ill Adults With Acute Respiratory Failure and Interstitial Lung Disease. Chest.

[3] Bradley B (2008). Interstitial lung disease guideline: the British Thoracic Society in collaboration with the Thoracic Society of Australia and New Zealand and the Irish Thoracic Society. Thorax.

[4] Travis WD (2013). An official American Thoracic Society/European Respiratory Society statement: update of the international multidisciplinary classification of the idiopathic interstitial pneumonias. Am. J. Respir. Crit. Care Med..

[5] Hansell DM (2008). Fleis- chner society: glossary of terms for thoracic imaging. Radiology.

[6] Akira M, Inoue Y, Arai T, Okuma T, Kawata Y (2011). Long-term follow-up high-resolution CT findings in non-specific interstitial pneumonia. Thorax.

[7] Silva CIS (2008). Nonspecific interstitial pneumonia and idiopathic pulmonary fibrosis: changes in pattern and distribution of disease over time. Radiology.

[8] Kosaka J (2013). Effects of biliverdin administration on acute lung injury induced by hemorrhagic shock and resuscitation in rats. PLoS One.

[9] Ranieri VM (2012). Acute respiratory distress syndrome: the Berlin Definition. Jama.

[10] Kotani T (2017). Risk Factors for the Mortality of Pneumocystis jirovecii Pneumonia in Non-HIV Patients Who Required Mechanical Ventilation: A Retrospective Case Series Study. Biomed Res Int.

[11] Gungor G (2013). Why do patients with interstitial lung diseases fail in the ICU? a 2-center cohort study. Respir Care.

[12] Gaudry S (2014). Invasive mechanical ventilation in patients with fibrosing interstitial pneumonia. J Thorac Cardiovasc Surg.

[13] Mollica C (2010). Mechanical ventilation in patients with end-stage idiopathic pulmonary fibrosis. Respiration.

[14] Oda K (2016). Efficacy of concurrent treatments in idiopathic pulmonary fibrosis patients with a rapid progression of respiratory failure: an analysis of a national administrative database in Japan. BMC Pulm Med.

[15] Kawamura K, Ichikado K, Suga M, Yoshioka M (2014). Efficacy of azithromycin for treatment of acute exacerbation of chronic fibrosing interstitial pneumonia: a prospective, open-label study with historical controls. Respiration.

[16] Oikonomou A (2014). Role of imaging in the diagnosis of diffuse and interstitial lung diseases. Curr Opin Pulm Med.

[17] Debray MP (2015). Interstitial lung disease in anti-synthetase syndrome: initial and follow-up CT findings. Eur J Radiol.

[18] Juhl KS, Bendstrup E, Rasmussen F, Hilberg O (2015). Emphysema mimicking interstitial lung disease: Two case reports. Respir Med Case Rep.

[19] Ichikado K (1997). Acute interstitial pneumonia: high-resolution CT findings correlated with pathology. AJR Am J Roentgenol.

